# Fat traffic control: S-acylation in axonal transport

**DOI:** 10.1016/j.molpha.2025.100039

**Published:** 2025-04-16

**Authors:** Amelia H. Doerksen, Nisandi N. Herath, Shaun S. Sanders

**Affiliations:** Department of Molecular and Cellular Biology, College of Biological Sciences, University of Guelph, Ontario, Canada

**Keywords:** fast axonal transport, glycolysis, molecular motor proteins, neurological disorders, palmitoylation, S-acylation

## Abstract

Neuronal axons serve as a conduit for the coordinated transport of essential molecular cargo between structurally and functionally distinct subcellular compartments via axonal molecular machinery. Long-distance, efficient axonal transport of membrane-bound organelles enables signal transduction and neuronal homeostasis. Efficient axonal transport is conducted by dynein and kinesin ATPase motors that use a local ATP supply from metabolic enzymes tethered to transport vesicles. Molecular motor adaptor proteins promote the processive motility and cargo selectivity of fast axonal transport. Axonal transport impairments are directly causative or associated with many neurodegenerative diseases and neuropathologies. Cargo specificity, cargo-adaptor proteins, and posttranslational modifications of cargo, adaptor proteins, microtubules, or the motor protein subunits all contribute to the precise regulation of vesicular transit. One posttranslational lipid modification that is particularly important in neurons in regulating protein trafficking, protein-protein interactions, and protein association with lipid membranes is S-acylation. Interestingly, many fast axonal transport cargos, cytoskeletal-associated proteins, motor protein subunits, and adaptors are S-acylated to modulate axonal transport. Here, we review the established regulatory role of S-acylation in fast axonal transport and provide evidence for a broader role of S-acylation in regulating the motor-cargo complex machinery, adaptor proteins, and metabolic enzymes from low-throughput studies and S-acyl-proteomic data sets. We propose that S-acylation regulates fast axonal transport and vesicular motility through localization of the proteins required for the motile cargo-complex machinery and relate how perturbed S-acylation contributes to transport impairments in neurological disorders.

**Significance Statement:**

This review investigates the regulatory role of S-acylation in fast axonal transport and its connection to neurological diseases, with a focus on the emerging connections between S-acylation and the molecular motors, adaptor proteins, and metabolic enzymes that make up the trafficking machinery.

## 1 Introduction

Neurons are large, complex cells with comparatively short input and long output projections known as dendrites and axons, respectively. Neurons receive information from other neurons at neuron-neuron connections known as synapses and propagate these signals to downstream targets along axons through electrical impulses called action potentials. The tightly controlled and distinct subcellular compartmentalization within neurons is crucial for neuronal activity and function, requiring efficient, organized trafficking of proteins and organelles to specific sites throughout these large, morphologically complex cells.

## 2 Brief historical perspective

Efficient and precise trafficking of cellular components is of particular importance in axons that can be up to a meter long in corticospinal tracts and the sciatic nerve (Cavanagh, 1984). Fast axonal transport overcomes the unique morphological challenge of transporting cargo over great distances between the cell body, called the soma, and axon tips. Fast, efficient transport involves the movement of diverse cargo along microtubules by molecular motor ATPases, at speeds reaching up to 40 cm/d (Griffin et al, 1976; Maday et al, 2014). This transport speed is achieved by the processive, unidirectional, continuous movement of molecular motors along microtubule highways. In axons, microtubules are uniformly oriented with plus ends toward axon tips and minus ends toward the soma (Burton and Paige, 1981; Stepanova et al, 2003; Stone et al, 2008). The directionality of transport carried out by the molecular motors is thereby defined by the polarity of the axonal microtubules, leading to the motors consistently facilitating transport in specific directions along axons (Vale et al, 1985; Paschal et al, 1987; Schroer et al, 1989). Kinesin motors move toward microtubule plus ends at axon tips to mediate anterograde transport, whereas cytoplasmic dynein 1 (hereby referred to as dynein) motors move cargo toward minus ends oriented toward the soma to drive retrograde transport (Hirokawa et al, 1990, 1991). Many cargos have an ensemble of motors with both types attached, with multiple levels of regulation to balance axonal cargo directionality and speed. Cargo trafficked via fast axonal transport includes synaptic and dense core vesicles carrying neurotransmitters and neurotrophins, late and signaling endosomes and lysosomes, mRNA granules, autophagosomes, mitochondria, and prodegenerative signaling complexes (reviewed in Hirokawa et al, 2010). Axonal growth, synaptic plasticity and neurotransmission, neuronal homeostasis and signaling, and distal axon survival are critically dependent on efficient axonal transport via the kinesin and dynein molecular ATPase motors (Maday et al, 2014).

Impaired axonal transport has been either directly or indirectly implicated in several neurodegenerative and neurological disorders clearly demonstrating the physiological importance of efficient axonal transport (Guo et al, 2020; Berth and Lloyd, 2023; Yang et al, 2023; Xiong and Sheng, 2024). Direct, genetic evidence comes from mutations in genes coding for motor proteins and their adaptors and regulators as well as cytoskeleton and associated proteins causing neurological diseases. These diseases include the neurodegenerative diseases Huntington, Parkinson, and Alzheimer diseases (HD, PD, and AD, respectively), amyotrophic lateral sclerosis, and spinal muscular atrophy, as well as hereditary spastic paraplegias, Perry syndrome, and hereditary motor and sensory neuropathies (recently reviewed in Guo et al, 2020; Berth and Lloyd, 2023; Yang et al, 2023; Xiong and Sheng, 2024).

The kinesin superfamily is diverse with 45 genes (*KIFs*) in the human genome (Miki et al, 2001). The general kinesin domain structure includes the motor domain, coiled-coil domain, and tail. The head motor domains associate with microtubules, and the coiled-coil domains form the filamentous stalk during dimerization. The kinesin tail binds to adaptor proteins, light chains, or directly to cargo. KIFs function as monomers or as dimeric or trimeric complexes with kinesin associated proteins and other adaptor protein scaffolds (Hirokawa et al, 1989; Yamazaki et al, 1996; Diefenbach et al, 1998; Miki et al, 2001; Blasius et al, 2007). The dynein motor is a ∼1.4 MDa multisubunit complex comprised of dynein heavy chains, intermediate chains, light intermediate chains, and light chains. The dynein tail, which consists of the intermediate chains, light intermediate chains, and light chains, interacts with adaptor proteins and cargo as well as the dynein cofactor, dynactin (Gill et al, 1991; Schroer and Sheetz, 1991; Pfister et al, 2005). Dynactin is a key activator protein complex required for many dynein functions including cargo-dynein interaction (Schroer, 2004; Zhang et al, 2017). Adaptor proteins mediate attachment of molecular motors to cargos, with some adaptors having an activating function to promote dynein processivity (Olenick and Holzbaur, 2019). Processive dynein-dynactin complex motility is stimulated by dynein adaptors such as bicaudal D2 (BICD2) and hook microtubule tethering protein 3 (HOOK3), which increase the dynein-dynactin interaction (Splinter et al, 2012; McKenney et al, 2014; Schlager et al, 2014; Olenick et al, 2016; Schroeder and Vale, 2016). Dynactin promotes faster dynein movement, processivity, and force production by recruiting dynein motors to dynactin-BICD2 or dynactin-HOOK3 complexes (Grotjahn et al, 2018; Urnavicius et al, 2018).

Fast axonal transport is not just dependent on the motor proteins and adaptors, but it also requires a readily available source of ATP as kinesin and dynein hydrolyze one ATP to fuel every 8 nm step (Hua et al, 1997; Schnitzer and Block, 1997; Mallik et al, 2005). Although oxidative phosphorylation produces significantly more ATP than glycolysis and meets most of the energy requirements in neurons (Mayevsky and Chance, 1975; Ames, 2000; Bonvento and Bolaños, 2021), mitochondria are concentrated to axonal regions with high energy demands such as synapses, growth cones, and nodes of Ranvier (Hollenbeck and Saxton, 2005; Chang et al, 2006; Tosolini et al, 2024). As such, mitochondrial energy production alone is unsuitable for meeting the constant energy demands of fast axonal transport throughout the length of the axon (Zala et al, 2013). Importantly, all 10 glycolytic enzymes tether to vesicular fast axonal transport cargo where they fulfill the minimum energy demands required to fuel molecular motors (Zala et al, 2013; Hinckelmann et al, 2016). Glycolysis is a 10-step cytosolic metabolic cascade that ultimately converts 1 glucose molecule into 2 pyruvate molecules, the final product of glycolysis, and involves the net production of 2 ATP molecules. Chemical inhibition or knockdown of the sixth glycolytic enzyme, glyceraldehyde 3-phosphate dehydrogenase (GAPDH) that oxidizes glyceraldehyde 3-phosphate, generating NADH from NAD+, impedes the movement of fast axonal transport cargo along microtubules, while inhibition of mitochondrial ATP production does not (Zala et al, 2013). Taken together, fast axonal transport is dependent on a local population of glycolytic machinery tethered to the motile cargo to meet the ATP demands of molecular motors to fuel transport.

For ATP production to occur continuously via glycolysis, metabolically favorable and energetically homeostatic conditions are required, in particular a high NAD+:NADH ratio and the clearing of pyruvate. Neurons rely heavily on mitochondrial oxidative phosphorylation over glycolysis as well as on the astrocyte-neuron lactate shuttle in vivo (Mayevsky and Chance, 1975; Knull, 1980; Maher et al, 1991; Pellerin and Magistretti, 1994; Ames, 2000; Bonvento and Bolaños, 2021; Li et al, 2023). This pathway involves the release of lactate by glycolytically active astrocytes, which is then taken up by neurons, converted to pyruvate, and finally consumed by the citric acid cycle to generate precursors for oxidative phosphorylation in neuronal mitochondria (Bonvento and Bolaños, 2021). This phenomenon requires lactate dehydrogenase (LDH), an enzyme that catalyzes the conversion between pyruvate and lactate, as well as NADH and NAD+ (Flick and Konieczny, 2002). There are 2 LDH isoforms, LDHA and LDHB (Markert et al, 1975). LDHA has a high affinity for pyruvate and catalyzes its conversion to lactate as well as NADH to NAD+, whereas LDHB catalyzes the reverse reaction (Flick and Konieczny, 2002; Chen et al, 2024). Importantly, astrocytes have high levels of LDHA, whereas neurons have more abundant LDHB, permitting lactate production from pyruvate in glycolytically active astrocytes followed by conversion back to pyruvate in neurons (Bittar et al, 1996; Mc Cluskey et al, 2024). However, in the unique context of fueling fast axonal transport, LDHA rather than LDHB plays a critical role (Mc Cluskey et al, 2024). Recently, the Saudou group found that both LDH isozymes localize to fast moving vesicles, but LDHA is more abundant (Mc Cluskey et al, 2024). Additionally, they ascertained that vesicular LDHA activity is critical for on-board fast axonal transport glycolysis and subsequent anterograde and retrograde transport kinetics. LDHA plays a crucial role for continual glycolysis during fast axonal transport by clearing pyruvate and regenerating NAD+ from NADH produced vesicle-bound GAPDH, thus sustaining the autonomous metabolic system that fuels fast axonal transport (Mc Cluskey et al, 2024).

**Fig. 1:**
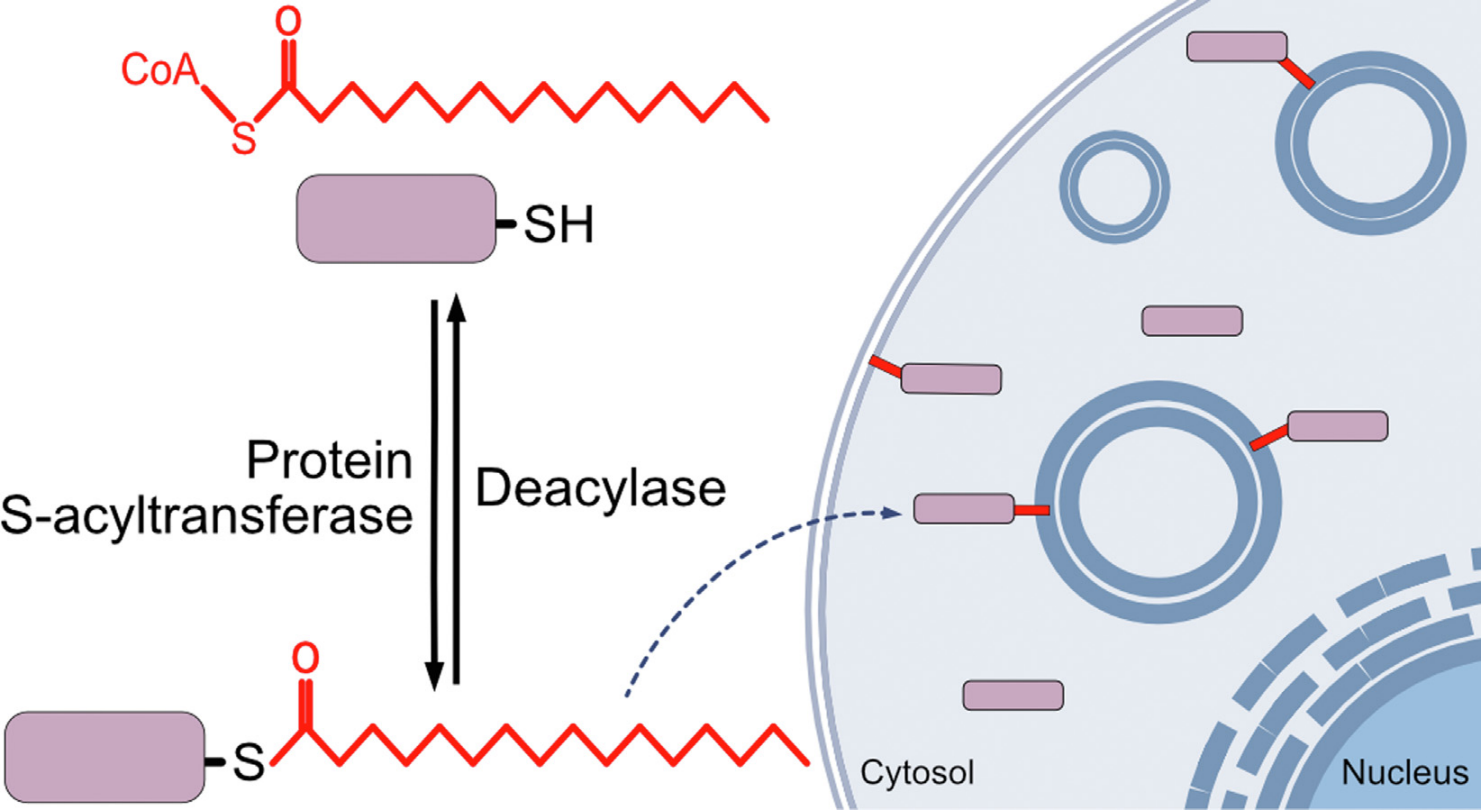
Protein S-acylation. Cysteine (-SH) residues of proteins are modified by LCFAs (red; here the 16-carbon saturated palmitate) by protein S-acyltransferases and deacylated by various deacylases, including acyl protein thioesterases, palmitoyl protein thioesterases, and *α*/*β* hydrolase domain-containing serine hydrolases. S-acylation regulates membrane affinity and protein localization and/or function.

Regulation of the various transported motile cargo is based on the cargo specificity of the motors involved, the association of adaptors that link cargo to motors, and posttranslational modifications of adaptors or motor proteins themselves (Morfini et al, 2002; Colin et al, 2008; Guillaud et al, 2008; Pineda et al, 2009; Vagnoni et al, 2011; Wang et al, 2011; Chua et al, 2012; Pathak et al, 2018; Banerjee and Gunawardena, 2023). A posttranslational lipid modification of increasing neuronal interest, including for fast axonal transport, is protein S-acylation. S-acylation is the reversible addition of a long-chain fatty acid (LCFA) to cysteine residues via a thioester bond (Fig. 1) (Magee and Courtneidge, 1985; Hallak et al, 1994; Mesquita et al, 2024b). S-acylation targets and regulates membrane association of many proteins in neurons. This includes scaffold proteins such as Ankyrin-G and Gephyrin as well as postsynaptic density 95 and 93 to recruit ion channels and receptors to the membrane at various locations, including the axon initial segment, nodes of Ranvier, and synapses (Topinka and Bredt, 1998; El-Husseini et al, 2000; He et al, 2012; Dejanovic et al, 2014; Tseng et al, 2015; Sanders et al, 2020; Petropavlovskiy et al, 2021). In the brain, the most common LCFAs used for S-acylation are saturated and monounsaturated fatty acids with 16- or 18-carbons, making up approximately 75% of the LCFAs used, with minor contributions from 20- and 22-carbon polyunsaturated LCFAs (Busquets-Hernández et al, 2024). Broadly, the 16-carbon saturated fatty acid palmitate is the most common LCFA in cells and, as such, this modification is commonly known as S-palmitoylation or, simply, palmitoylation (Mesquita et al, 2024a). S-acylation is mediated by protein S-acyltransferases, belonging to a family of 23 zinc finger and DHHC (aspartate-histidine-histidine-cysteine) catalytic motif-containing enzymes (ZDHHC1-24, skipping 10 in humans), whereas removal of LCFAs is facilitated by several classes of deacylases including acyl protein thioesterases (APTs), palmitoyl protein thioesterases, and *α*/*β* hydrolase domain proteins (Fig. 1) (Putilina et al, 1999; Mesquita et al, 2024a; Wang et al, 2024).

In silico estimates suggest that S-acylation may be the fifth most common posttranslational modification (Khoury et al, 2011). Indeed, S-acylation is the most common protein lipid modification in the brain with 10%–20% of the genome coding for a proteoform that can be S-acylated (Fukata and Fukata, 2010; Blanc et al, 2015; Sanders et al, 2015; Mesquita et al, 2024a) and mutations in or loss of several *ZDHHC* genes result in predominantly neuronal phenotypes (Mukai et al, 2004, 2008, 2015; Mansouri et al, 2005; Raymond et al, 2007; Li et al, 2010; Singaraja et al, 2011; Sutton et al, 2013; Baker et al, 2015; Kilpatrick et al, 2016; Sanders et al, 2016; Mejias et al, 2021; Mesquita et al, 2024a). Although almost 50% of the synaptic proteome is S-acylated and a lot is known regarding the regulation of synaptic protein localization (Petropavlovskiy et al, 2021), much is still to be explored regarding the role of S-acylation in regulating the axonal trafficking machinery itself. Several fast axonal transport cargo, cytoskeletal-associated proteins, and motor protein subunits and adaptors have been shown to be S-acylated, which regulates their axonal localization and transport. Increasing the interest in S-acylation in the context of neuronal trafficking, a pivotal study by the Saudou group showed that restoring S-acylation by pharmacological inhibition of the deacylating enzyme APT1 in the brain of HD mice is protective and restores trafficking deficits in cultured HD cells (Virlogeux et al, 2021).

In this review, we will discuss the known roles of S-acylation in regulating fast axonal transport in neurons, relating to the S-acylation of molecular motor complexes, cargo, and metabolic enzymes, and the impacts of S-acylation impairments on transport deficiencies and subsequent neurodegeneration and neurological disorders. In addition, we provide further evidence from S-acyl-proteomic studies for S-acylation of dynein and kinesin motor protein subunits and the dynein cofactor dynactin as well as for key dynein adaptor proteins and metabolic enzymes present on fast transport vesicles, suggesting that S-acylation may play a broader role in regulating fast axonal transport.

## 3 Key recent advances

To date, there is only one motor protein subunit known to be S-acylated. The cytoplasmic dynein 1 intermediate chain 1 (DYNC1I1) was identified in an S-acyl proteomic study from cultured primary rat cortical neurons and rat whole brain and was subsequently verified in low-throughput experiments (Kang et al, 2008). However, no further studies have been done to determine the functional role of S-acylation of DYNC1I1, an important outstanding question.

There are several motor protein adaptors known to be S-acylated. The integral lysosomal protein transmembrane protein 55B (TMEM55B; also known as phosphatidylinositol-4,5 bisphosphate 4-phosphatase) is a cargo adaptor that recruits the dynein-dynactin complex to lysosomes by interaction with the cargo scaffolding protein c-Jun N-terminal kinase (JNK)-interacting protein 4 (JIP4) (Willett et al, 2017). TMEM55B is S-acylated and its S-acylation is required for export from the Golgi and sorting to lysosomes in non-neuronal cells, potentially by facilitating interaction with other adaptors (Rudnik et al, 2022). However, it is unknown whether the same mechanism occurs in neurons to regulate lysosomal sorting to axons, or if S-acylation regulates lysosomal positioning through the JIP4 and TMEM55B interaction, or cargo vesicle sorting for anterograde transport.

Glutamate receptor interacting protein 1 (GRIP1) is an adaptor that interacts with kinesin heavy chains (KIF5) in neurons to direct kinesin motor driven endosomes carrying AMPA (*α*-amino-3-hydroxy-5-methylisoxazole-4-propionate) glutamate receptors into dendrites (Setou et al, 2002). The GRIP1b isoform is S-acylated and S-acylation targets GRIP1b to dendritic endosomes and regulates binding to KIF5 (Thomas et al, 2012). Although this is an example of regulation for a dendritic sorting motor protein adaptor, it highlights the potential relevance of S-acylation in modulating other adaptor-motor protein interactions to control vesicular transport into axons.

The LIS1-NDEL1-NDE1 (Lissencephaly 1, Nuclear distribution protein nudE-like 1, and nudE neurodevelopment protein 1, respectively) complex is involved in dynein intracellular transport activity. LIS1, NDEL1, and NDE1 interact with dynein (Niethammer et al, 2000; Sasaki et al, 2000) and participate in regulating dynein-mediated transport (Yi et al, 2011; Pandey et al, 2022; Zhao et al, 2023). NDEL1 and NDE1 are S-acylated in neurons, and NDEL1 S-acylation negatively regulates its interaction with the dynein complex and decreases retrograde dynein trafficking. In contrast, NDE1 S-acylation had no impact on its interaction with dynein. Interestingly, the differential effects of NDE1 and NDEL1 S-acylation on their interactions with dynein may be due to differences in the amino acids surrounding the S-acylation site that may mediate protein-protein interactions, but this warrants further investigation (Shmueli et al, 2010).

Huntingtin (HTT), the protein mutated in HD, is an adaptor for kinesin and dynein to either directly or indirectly link these motors to various cargo and cargo-adaptor complexes in neurons (Engelender et al, 1997; Li et al, 1998; Gauthier et al, 2004; Caviston et al, 2007; Vitet et al, 2020, 2023; Cason et al, 2021). This function of HTT is impaired when it is mutated in HD (Gauthier et al, 2004; Twelvetrees et al, 2010; Yu et al, 2018; Vitet et al, 2020). HTT is also S-acylated at multiple cysteines by ZDHHC13 and ZDHHC17, and its S-acylation is important for its neuronal localization and, when mutated, its S-acylation is impaired, which is associated with mutant HTT aggregation and neuronal toxicity (Huang et al, 2004, 2009, 2011; Yanai et al, 2006; Singaraja et al, 2011; Lemarié et al, 2021, 2023). However, whether S-acylation may serve as a tethering mechanism to bind HTT to fast axonal transport vesicles to regulate its adaptor function and if impaired HTT S-acylation in HD contributes to trafficking deficits is unknown. Importantly, the Hayden lab found that overexpressing the HTT S-acylating enzyme ZDHHC17 or pharmacological inhibition of the HTT deacylating enzymes APT1 and APT2 increases mutant HTT S-acylation, reduces its aggregation, and rescues neurotoxicity in multiple cellular models, including neurons from HD mouse models (Lin and Conibear, 2015; Lemarié et al, 2021). Further work by the Saudou group showed that pharmacological inhibition of APT1 restores axonal trafficking in cultured mouse and human induced pluripotent stem cell derived HD neurons and rescues neuropathology and behavioral deficits in HD mice (Virlogeux et al, 2021). These are exciting findings demonstrating the potential utility of pharmacological manipulation of S-acylation to treat neurodegenerative disorders associated with impaired axonal trafficking.

In addition to motor protein adaptors, other regulators of axonal transport, including microtubule-associated proteins, axon protective factors, and axonal cargo, are modulated by S-acylation, demonstrating that S-acylation plays a role in coordinating the microtubule-dependent processes that govern axonal trafficking. S-acylation regulates 2 microtubule-associated proteins including Stathmin-2 (STMN2, also known as superior cervical ganglion 10) and microtubule-associated protein 6 (MAP6) (Gory-Fauré et al, 2006, 2014; Levy et al, 2011; Tortosa et al, 2017). STMN2 is involved in maintaining axonal integrity and loss of STMN2 is associated with amyotrophic lateral sclerosis and various neuropathies (Klim et al, 2019; Melamed et al, 2019; Thornburg-Suresh et al, 2023). STMN2 S-acylation is required for its vesicle association and its axon protection effects (Thornburg-Suresh et al, 2023) but not its interaction with tubulin nor its ability to regulate microtubule stability (Di Paolo et al, 1997; Lutjens et al, 2000; Levy et al, 2011). S-acylation also targets STMN2 for mitogen-activated protein kinase (MAPK) signaling-dependent degradation (Summers et al, 2018). Interestingly, following nerve growth factor application, S-acylated and phosphorylated STMN2 degradation is increased via a phospholipase C-dependent pathway, which increases Wallerian degeneration, namely degeneration distal to the axon injury site from the soma. This suggests a role for S-acylation in regulating STMN2 proteostasis and Wallerian degeneration in response to nerve growth factor-mediated signaling (Danos et al, 2025).

MAP6 is enriched in axons where it stabilizes microtubules (Bosc et al, 1996; Guillaud et al, 1998), and its S-acylation is required for binding to secretory vesicles and sorting to the axon (Tortosa et al, 2017). MAP6 promotes KIF5B-driven axonal cargo transport, like other MAPs that regulate motor protein motility (Barlan et al, 2013; Tortosa et al, 2017; Chaudhary et al, 2019; Monroy et al, 2020). Altered MAP6 S-acylation could, in turn, affect KIF5B transport dynamics. Interestingly, pathogenic *α*-synuclein disrupts MAP6 S-acylation and pharmacological inhibition of APT1 increases MAP6 S-acylation and protects against *α*-synuclein-dependent neurotoxicity, providing an interesting mechanistic link between S-acylation and altered *α*-synuclein-dependent vesicular transport that is characteristic of synucleinopathies such as PD (Ho et al, 2020). This is another intriguing demonstration of the potential for pharmacological targeting of S-acylation to treat neurodegenerative diseases.

Nicotinamide mononucleotide adenylyltransferase-2 (NMNAT2), an axon protective factor that synthesizes NAD+ from its substrate nicotinamide mononucleotide (NMN), is also S-acylated (Mayer et al, 2010; Milde and Coleman, 2014). Broadly, the sustained presence of NMNAT2 in distal axons, resulting from its constant anterograde trafficking, is important for axonal growth during development and axonal integrity throughout life (Lau et al, 2010; Mayer et al, 2010; Gilley et al, 2019; Niu et al, 2020). Most importantly, NMNAT2 maintains a high NAD+:NMN ratio that is prosurvival in axons by countering the NAD-degradation enzyme sterile alpha and TIR motif-containing protein 1 (SARM1) (Osterloh et al, 2012; Figley et al, 2021; Yang et al, 2024). If NMNAT2 delivery to the distal axon is halted, such as following axonal injury, SARM1-mediated axon degeneration, known as Wallerian degeneration, is triggered by a high NMN:NAD+ ratio (Milde and Coleman, 2014; Yang et al, 2024). NMNAT2 is S-acylated at adjacent cysteine residues C164 and C165, which, together with surrounding basic residues, is required for its association to Golgi-derived and axonally trafficked vesicles (Milde et al, 2013). Interestingly, loss of the NMNAT2 S-acylating enzyme ZDHHC17 in cultured rat dorsal root ganglion neurons resulted in distal axon degeneration, suggesting NMNAT2 S-acylation is critical for its anterograde trafficking and ability to maintain axonal integrity (Niu et al, 2020). Conversely, S-acylated NMNAT2 is more susceptible to MAPK signaling-dependent degradation than an S-acyl-deficient variant of NMNAT2 (Summers et al, 2018).

Furthermore, reduced anterograde and retrograde vesicle motility as well as impaired glycolytic ATP production have been observed in distal axons of NMNAT2 knockout neurons. This suggests a role, in addition to its primary function of blocking SARM1-mediated degeneration, for NMNAT2 in maintaining NAD redox potentials necessary to metabolically support fast axonal transport (Yang et al, 2024). In the context of maintaining a favorable NAD+:NADH ratio for continuous vesicular glycolysis, NMNAT2 synthesizes NAD+ and is present on fast axonal transport vesicles (Mayer et al, 2010; Milde and Coleman, 2014), which may be important for providing substrates for GAPDH (Yang et al, 2024). It would be interesting to assess whether blocking NMNAT2 S-acylation may hinder vesicular glycolysis in the distal axon in a similar fashion to NMNAT2 knockout. However, the role of NMNAT2 S-acylation in supporting the metabolic requirements of fast axonal transport in the distal axon has yet to be elucidated.

Finally, ankyrin-B (ANKB, coded by the gene *ANK2*) is a scaffold protein that promotes fast axonal transport by serving as a link between organelle cargo and the dynein-dynactin complex (Lorenzo et al, 2014). ANKB is S-acylated in brain tissue, but, interestingly, its S-acylation does not regulate ANKB-mediated transport in axons, and synaptic vesicle axonal trafficking is not dependent on its S-acylation state; instead, ANKB S-acylation is necessary for scaffolding of the dendritic voltage-gated sodium channel Na_v_1.2 (Gupta and Jenkins, 2023). Despite ANKB S-acylation not seeming to have a direct modulatory role in ANKB-mediated axonal transport (Gupta and Jenkins, 2023), S-acylation is still critical for regulating axonal transport for other key neuronal proteins and it is important to still examine potential S-acylation-mediated axonal transport dynamics for other S-acylated or potentially S-acylated proteins.

Additionally, there are several key fast axonal cargos that are dependent on S-acylation for transport in the axon. Dual leucine zipper kinase (DLK; MAP3K10) and JNK3 (also known as MAPK10) are kinases in the MAPK signaling pathway involved in retrograde, prodegenerative responses in neurons (Niu et al, 2020, 2022). Following axonal injury, DLK, a MAP3K, activated proximal to the injury, will phosphorylate MAPK kinase 4 and MAPK kinase 7, which will then phosphorylate JNK3, which, finally, will phosphorylate c-Jun, a transcription factor involved in upregulating somal responses to axonal injury (Fan et al, 1996; Hammarlund et al, 2009). However, for somal activation of c-Jun to occur, DLK axon to soma signaling is required. Importantly, S-acylation of DLK and JNK3 is required for their trafficking via retrograde fast axonal transport after axonal injury to activate somal prodegenerative responses (Holland et al, 2016; Niu et al, 2020, 2022). Interestingly, S-acylation is required for DLK kinase activity and permits its further activation by JNK3 in a positive feedback loop (Holland et al, 2016; Niu et al, 2022). In addition to DLK/JNK3, another retrograde signaling complex relies on S-acylation (Collura et al, 2020). Glycoprotein 130 (Gp130; also known as interleukin-6 receptor subunit beta) is a neuropoietic cytokine receptor that activates the Janus kinase/signal transducer and activator of transcription pathway (Bauer et al, 2007). S-acylation of Gp130 by ZDHHC5 and ZDHHC8 is required for its surface expression in axons and retrograde signaling (Collura et al, 2020).

## 4 Current challenges and knowledge gaps

**Fig. 2:**
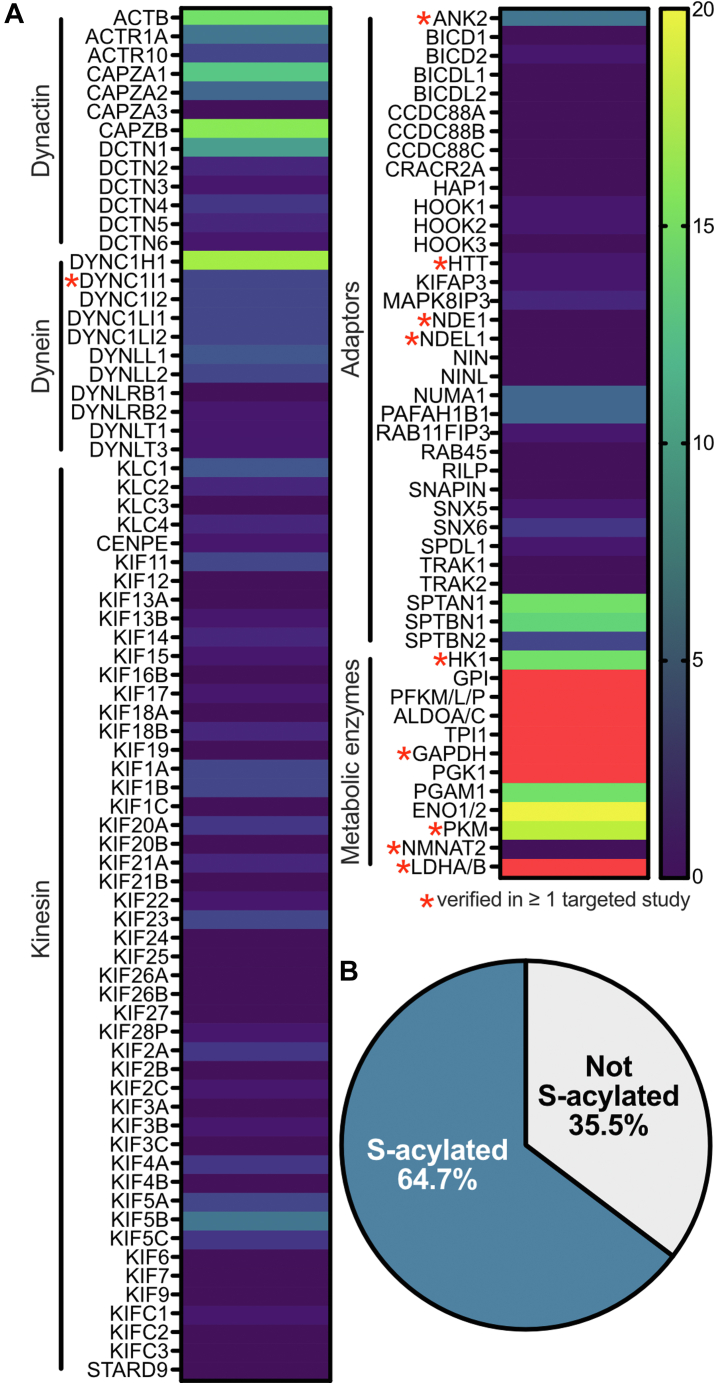
Comparison of a list of trafficking machinery to S-acyl proteomic studies reveals an enrichment of S-acylation. (A) Heat maps showing the number of S-acyl proteomic studies in which the trafficking machinery were identified. The list includes dynactin, dynein, and kinesin protein subunits, motor protein adaptors, and metabolic enzymes that fuel transport while bound to fast axonal trafficking vesicles. The gene set was compared to data from 48 human, mouse, and rat S-acyl proteomic studies using the Swisspalm database (https://swisspalm.org). Red indicates values above 20 and a red star indicates proteins were verified as S-acylated in at least one low throughput study. (B) Venn diagram depicting the percentage of S-acylated and non-S-acylated proteins for the trafficking machinery dataset. Proteins identified in at least one S-acyl-proteomic study or targeted study were counted as S-acylated.

To further explore the importance of S-acylation in regulating fast axonal trafficking, we sought to determine if S-acylation is a prominent modification of axonal transport machinery. To do so, we generated a list of kinesin and dynein motor protein subunits as well as subunits of the essential dynein cofactor complex, dynactin, and several key dynein adaptor proteins to upload to the SwissPalm S-acylated protein database and compare to all available human, mouse, and rat S-acyl proteomic datasets (48 in total; Blanc et al, 2015). Motor protein subunit genes were retrieved from the HUGO Gene Nomenclature Committee (genenames.org) using keywords “kinesin,” “dynein 1,” and “dynactin” to identify the following HGNC gene groups: kinesin (*KIF*), dynein 1 complex subunits (*DYNC1*), and dynactin subunits (*DCTN*) (Seal et al, 2023). No Uniprot entry was found for KIF4CP, so it was excluded from subsequent analyses. Dynein adaptor genes were pooled from 3 recent reviews and the kinesin adaptor *KIFAP3* was added manually, as was *ACTB* (actin beta), which is present within the dynactin filament of the dynactin complex (Yamazaki et al, 1996; Dupuis et al, 2000; Tateno et al, 2009; Urnavicius et al, 2015; Reck-Peterson et al, 2018; Olenick and Holzbaur, 2019; Canty and Yildiz, 2020). The resulting gene list was uploaded to Swisspalm to generate a list of trafficking machinery identified in S-acyl proteomic and/or low-throughput validation studies (Supplemental Table 1, including further methodological details). The number of S-acyl proteomic studies, in which each protein was identified, was plotted as heat maps, demonstrating the high proportion of kinesin, dynein, and dynactin subunits as well as dynein adaptors that are likely S-acylated (Fig. 2A). S-acylation may be regulating multiple aspects of motor protein function, including complex assembly, association with microtubules, and axonal trafficking dynamics, depending on the potential S-acyl sites. Our findings provide an intriguing area of future investigation to contribute foundational evidence of how S-acylation of motor proteins and adaptors regulates their trafficking and function.

With respect to the metabolic machinery required to energetically sustain fast axonal transport, what remains unclear is how the soluble, cytosolic, glycolytic enzymes tether to fast moving vesicles. The proposed mechanism for GAPDH tethering to vesicles is via HTT (Zala et al, 2013). However, loss of wild-type HTT minimally impacts fast axonal transport, and no mechanism was provided for vesicle tethering of the other glycolytic enzymes (Zala et al, 2013). We sought to determine if S-acylation may hold the answer. To do so, we included all 10 glycolytic enzymes and LDH, including isozymes, in our analysis described above and pulled S-acyl proteomic and low-throughput validation data from the Swisspalm database. Based on our analysis, the glycolytic enzymes and LDH have been identified in a striking number of S-acyl proteomic studies (Fig. 2A; Supplemental Table 1) (Blanc et al, 2015). As such, S-acylation appears to be an attractive tethering mechanism of interest to investigate in the context of metabolic enzymes required on fast axonal transport vesicles to fuel transport. As various metabolic enzymes, such as hexokinase (HK), GAPDH, pyruvate kinase (PK), and LDHA, are known to be S-acylated in other cell types, it would be fascinating to determine whether S-acylation of these enzymes occurs in neurons, and if S-acylation provides a specific or distinct functional role in this cellular context. It would be important to assess whether their S-acylation provides a mechanism for their targeting to fast axonal transport vesicle membranes, which would further our understanding of the unique metabolic system fueling fast axonal transport.

Importantly, HK, the first enzyme of the glycolytic pathway, and PK, the final rate-limiting and ATP producing glycolytic enzyme, as well as GAPDH have been identified as S-acylated using low-throughput methods (Yang et al, 2005; Chen et al, 2022; He et al, 2025). HK S-acylation is required for its targeting to the plasma membrane and secretion in extracellular vesicles by hepatic stellate cells, which contributes to hepatocellular carcinoma by upregulating glycolysis (Chen et al, 2022). Enhanced S-acylation of the M2 isoform of PK was found to contribute to palmitic acid-induced endothelial injury in the heart by impairing glycolysis (He et al, 2025). Meanwhile, GAPDH S-acylation was found to decrease its catalytic activity in vitro (Yang et al, 2005). In addition to the glycolytic enzymes, LDHA has been confirmed to be S-acylated in pancreatic cancer cells (Blanc et al, 2015; Chen et al, 2024). The S-acylation status in neurons and whether it provides a mechanism of targeting to fast axonal transport vesicle membrane for HK, GAPDH, PK, and LDHA has yet to be elucidated.

Proteins in our list of trafficking machinery, including motor proteins and their adaptors as well as metabolic enzymes tethered to fast axonal transport vesicles required for fueling transport, were counted as likely S-acylated if they were identified in one or more S-acyl proteomic and/or low-throughput validation studies. Interestingly, our analysis suggests that ∼65% of the trafficking machinery is likely to be S-acylated (Fig. 2B). These intriguing findings suggest that S-acylation may play an important, underappreciated role in regulating motor protein function and neuronal transport.

To determine if loss of S-acylation may directly lead to neuropathologies, we downloaded all pathogenic, likely pathogenic, and uncertain significance missense gene mutations from the ClinVar database (Landrum et al, 2014) for our list of likely S-acylated motor proteins, adaptors, and metabolic enzymes (identified in one or more studies from Supplemental Table 1). We then filtered the list of mutations for cysteine amino acid changes and manually determined if the cysteines are known or predicted to be S-acylated using the SwissPalm (Blanc et al, 2015) curated, high-threshold predictions from the CSS-Palm 4.0 S-acylation prediction program (Ren et al, 2008). Disease-associated gene mutations leading to amino acid changes in predicted S-acylation sites were identified in 4 genes including *KIF1A*, *KIF5A*, *BICD2*, and *ACTB* (Table 1). KIF1A isoform 1 C1277 (UniProt ID: Q12756-1) is predicted to be S-acylated, and mutations causing C1277Y or C1277G amino acid changes were identified in 2 patients with hereditary sensory and autonomic neuropathy type IIC, autosomal dominant 9 intellectual disability, and autosomal recessive spastic paraplegia 30. In addition to KIF1A, KIF5A C14 (isoform Q12840; UniProt ID: Q12840) is predicted to be S-acylated and a C14Y amino acid change was identified in an individual with spastic paraplegia. BICD2 C544 is predicted to be S-acylated in isoforms 1 and 2 (UniProt IDs: Q8TD16-1 and Q8TD16-2, respectively). A *BICD2* mutation leads to a C544Y amino acid change identified in a patient with autosomal dominant spinal muscular atrophy with contractures. Finally, ACTB (isoform P60709; UniProt ID: P60709) is predicted to be S-acylated at C272 and a Cys to Arg change at this site was associated with Baraitser-Winter syndrome 1. Determining if these disease-associated variants have impaired S-acylation and how that impacts axonal trafficking and contributes to disease is an interesting area of future research.

**Table 1:** Disease-associated cysteine amino acid changes in trafficking machinery at predicted S-acylation cysteine sites

Gene	Protein Change	Associated Condition	Predicted Cysteine(s)	Classification	Accession
*KIF1A*	C1277Y C1277G	Neuropathy, hereditary sensory, type 2C; intellectual disability, autosomal dominant 9; hereditary spastic paraplegia 30	1125, 1277	Uncertain significance	VCV000862867 VCV002149552
*KIF5A*	C14Y	Spastic paraplegia	14, 303, 304	Uncertain significance	VCV002851170
*BICD2*	C544Y	Autosomal dominant childhood-onset proximal spinal muscular atrophy with contractures	544	Uncertain significance	VCV000860417
*ACTB*	C272R	Baraitser-Winter syndrome 1	17, 272	Likely pathogenic	VCV003075720

## 5 Perspective on future directions

**Fig. 3:**
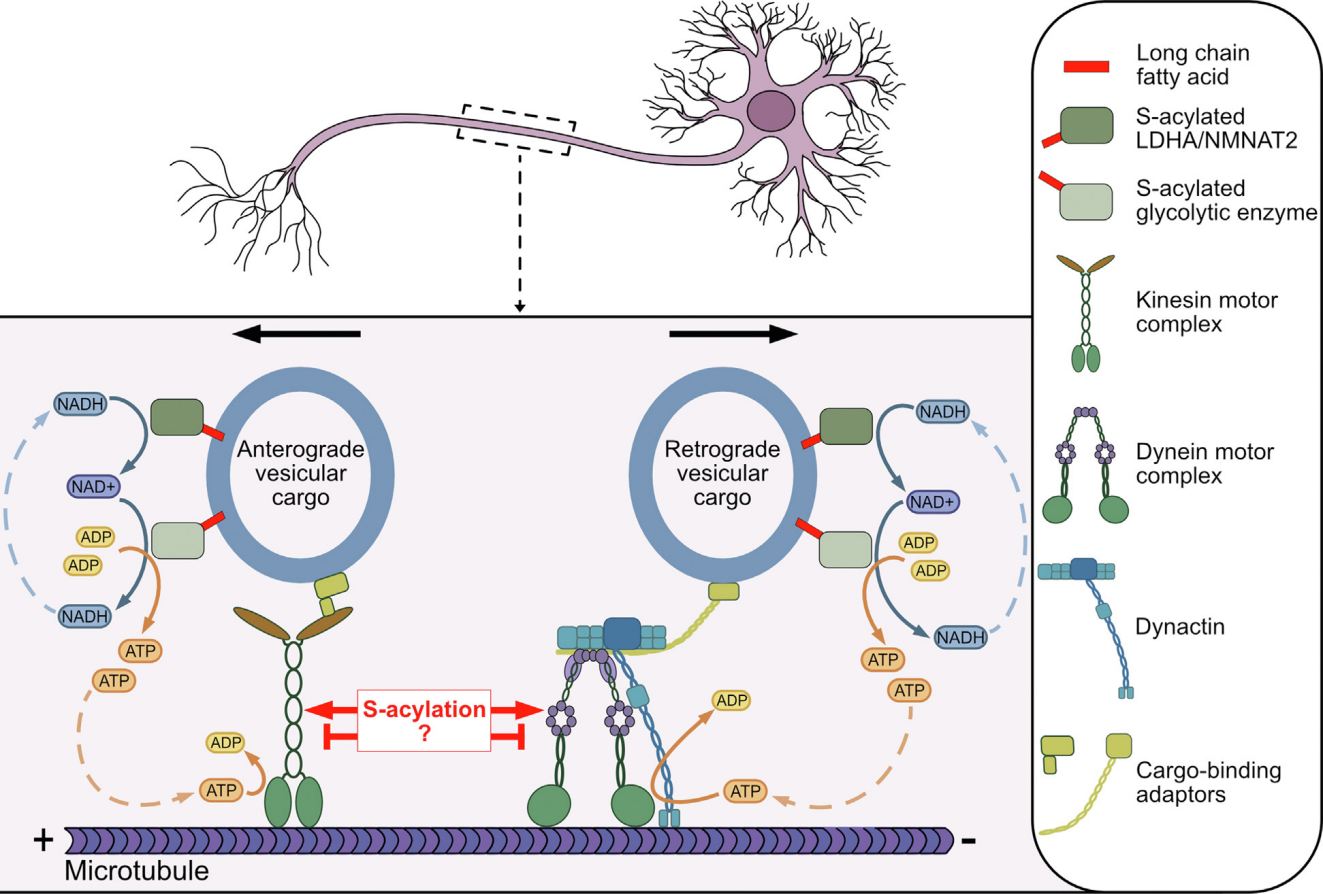
S-acylation-dependent regulation of axonal trafficking. Axons are long neuronal output projections in which microtubules are oriented with plus ends toward axon tips. Kinesin motors facilitate transport in the anterograde direction toward axon tips and dynein motors drive transport toward the soma in the retrograde direction (arrows indicate direction of transport). Glycolytic enzymes as well as LDHA and NMNAT2 are metabolic enzymes bound to transport vesicles to produce ATP locally to fuel transport. These metabolic enzymes are likely S-acylated and S-acylation may serve to tether them to membranous fast axonal transport vesicles to fuel transport (indicated by red bar). Many of the motor protein subunits and their adaptors are also likely S-acylated but how and when S-acylation regulates their function in axonal trafficking is unknown (unknown role indicated by “S-acylation?” and arrows or inhibition lines in red). S-acylation of the motor protein subunits and adaptors may negatively regulate trafficking or could be required for retention in cellular compartments or for protein-protein interactions potentially prior to or during axonal transport.

Only a small number of motor protein subunits or their adaptors have been confirmed as S-acylated and even less is known regarding the functional role of their S-acylation. Our new analysis and list of potentially S-acylated trafficking machinery provides an extensive basis for follow-up study (Fig. 2; Supplemental Table 1). Indeed, how S-acylation of motor protein subunits themselves may regulate their function is unclear (Fig. 3). As with the hypothesis that S-acylation serves to tether metabolic enzymes to fast transport vesicles to fuel transport, one possible hypothesis for a functional role of S-acylation of adaptor proteins is to bind them to the membranous cargo vesicles (Fig. 3). For example, sorting nexin 6 interacts with the dynactin subunit p150^Glued^/DCTN1 and coordinates microtubule-mediated dynein transport (Hong et al, 2009; Wassmer et al, 2009). The predicted sorting nexin 6 S-acylation site, C347 (isoform 1; UniProt ID: Q9UNH7-1) (Ren et al, 2008; Blanc et al, 2015), is within its BAR (Bin/Amphiphysin/Rvs) domain that interacts with membranes (McMahon and Gallop, 2005). S-acylation at this site may provide stable anchoring to the membrane and, in turn, aid in cargo transport. However, the story may be more complicated, as with NDEL1, where S-acylation may instead prevent interactions with the motor protein subunits and negatively regulate trafficking or may direct cargo sorting to specific motors or preferentially to either axons or dendrites. LIS1 and NDEL1 and the dynactin complexes move long distances along the axon while not associated with dynein (Fellows et al, 2024). Both LIS1 and NDEL1 localization at the neurite tip is reduced when NDEL1 S-acylation is blocked, hinting that NDEL1 S-acylation may serve to target LIS1 and NDEL1 to distal axons where they can then initiate dynein-mediated transport (Shmueli et al, 2010). This would likely require deacylation in the distal axon prior to binding to dynein. Indeed, MAP6 S-acylation sorts it to axons but it must be deacylated in the axon to bind microtubules (Tortosa et al, 2017). Thus, an intriguing hypothesis is that similar mechanisms may exist for motor protein adaptors and dynactin. It would be fascinating to determine if S-acylation is implicated in spatially regulating adaptors for transport away from the cell body before they associate together with the dynein-dynactin complex in distal axons.

It is also interesting to consider how the location of S-acylation sites within specific protein-protein or protein-microtubule interaction domains in motor protein subunits and adaptors might influence trafficking. Interestingly, one of the predicted p27/DCTN6 S-acylation sites, C18 (UniProt ID: O00399; Ren et al, 2008; Blanc et al, 2015), is within its face B that forms a dimer interface with p26/DCTN2 (Yeh et al, 2013). We would predict that S-acylation at this site would inhibit complex formation, as with NDEL1 S-acylation and dynein binding. Similarly, the predicted BICD2 site, C544 (isoforms 1 and 2, UniProt IDs: Q8TD16-1 and Q8TD16-2, respectively) (Ren et al, 2008; Blanc et al, 2015), is within its kinesin-1 interaction domain (Grigoriev et al, 2007; Splinter et al, 2010). Based on cryoelectron microscopy structures of the dynein-dynactin and kinesin complexes (Zhang et al, 2017; Urnavicius et al, 2018; Lau et al, 2021; Jiang et al, 2023), we predict that motor subunits and their predicted S-acylation sites are likely too far from the vesicle membrane for S-acylation to serve as an anchor to the vesicle. As such, we hypothesize that S-acylation of motor subunits negatively regulates trafficking, where perhaps S-acylation sequesters subunits away from microtubules or other components of the complex to a membrane microdomain or prevents complex formation by inhibiting protein-protein interactions. For many KIFs and cytoplasmic dynein 1 heavy chain 1, the predicted S-acylation sites are within their respective motor domains. In these examples, we would also predict that S-acylation would inhibit motor function and may allow for spatial or temporal regulation of the motor proteins prior to local deacylation and activation. The regulation of motor protein function may also vary with spatial distribution within the soma and axon as localization of S-acylating and deacylating enzymes is likely to influence timing and localization of S-acylation events to fine-tune trafficking complexes and cargo delivery. Overall, further insight into S-acylation of motor protein complexes and its impact on complex formation and function will allow assessment into the role of S-acylation in axonal trafficking.

The metabolic mechanisms that energetically fuel fast axonal transport diverge from metabolic strategies used to fuel the rest of the neuron, highlighting the level of specificity and complexity of energy dynamics within a single cell. Regarding fast axonal transport energy dynamics, there are many future avenues to explore, in addition to definitively assessing the S-acylation status of the key enzymes discussed in this review and determining if their S-acylation provides a mechanism to tether them to fast axonal transport vesicles (Fig. 3). It would be interesting to assess the impact S-acylation may have on vesicle-bound enzyme activity as well as the isozyme specificity of S-acylation. Vesicular glycolysis was found to be more efficient than that occurring in the cytosol in the brain (Mc Cluskey et al, 2024). It would be interesting to elucidate whether S-acylation of vesicular populations of glycolytic machinery may contribute to these differences in activity and efficiency. As LDHA and NMNAT2 play a similar role for fast axonal transport metabolism by replenishing NAD+ levels to fuel vesicular glycolysis, it will be important to determine whether LDHA and NMNAT2 colocalize or if they are found on distinct vesicle populations. Additionally, given the unique reversible nature of this lipid modification, it would be important to assess whether S-acylation of the metabolic enzymes is dynamic and how that may impact metabolism, transport kinetics, and glycolytic machinery turnover.

Finally, metabolic deficits and fast axonal transport impairments are well characterized in many neurodegenerative diseases (Chu et al, 2012; Hinckelmann et al, 2013; Manzo et al, 2019; Zamponi and Pigino, 2019; Cunnane et al, 2020; Bonvento and Bolaños, 2021). Given the increasingly important role S-acylation plays in neurons and the recent groundbreaking findings showing the neuroprotective effects of pharmacological upregulation of S-acylation in PD and HD models (Ho et al, 2020, 2023; Lemarié et al, 2021; Virlogeux et al, 2021), it will be important to explore whether loss of S-acylation may impair localization of motor proteins and their adaptors and/or the tethering of metabolic enzymes to fast axonal transport cargo. This may ultimately point to regulation of trafficking by S-acylation as contributing to neurodegenerative pathologies and its potential as a novel therapeutic target. The possibility of a direct causal role of cysteine amino acid changes in trafficking machinery in neurological disorders is also an intriguing area of future work, which could directly implicate aberrant S-acylation in related diseases. In addition, this analysis is not limited to loss of S-acylated cysteines, it would be interesting to also identify amino acid changes surrounding S-acyl cysteine sites that may also impact S-acylation and gain of cysteine changes that could be S-acylated ectopically.

## 6 Conclusions

Answering the questions proposed here will be critical for determining how S-acylation participates in establishing and modulating axonal transport dynamics and kinetics. Overall, our review and analysis suggest a broader role for S-acylation in regulating and fueling axonal trafficking machinery and provides a resource for further investigation and discovery of S-acylation-dependent protein targeting, regulation, and function in the axon.

## Conflict of interest

The authors declare no conflicts of interest.
