# Supplementary material for: Fat traffic control: S-acylation in axonal transport
Source: Mol Pharmacol. 2025 Apr 16;107(6):100039. doi: 10.1016/j.molpha.2025.100039 (PMC12264562; doi:10.1016/j.molpha.2025.100039)
Supplement: Supplementary Table 1 [file mmc1.pdf]

Fat traffic control: S-acylation in axonal transport. Amelia H Doerksen, Nisandi N Herath, and Shaun S Sanders. Molecular pharacology. Manuscript number: MOLPHA-D-24-00047R1.

Supplemental table 1: Motor protein subunits, adaptors, and metabolic enzyme S-acylation data from 22 human, 23 mouse, and 3 rat S-acyl-proteomic studies. If SwissPalm had no data for rat or mouse gene and the UniProt ID did not exist in SwissPalm, homologs were used if data was present. **Green indicates no mouse data in swisspalm for the reviewed gene or unreviewed homologs.** **Purple indicates no rat data in swisspalm for the reviewed gene or unreviewed homologs.** **Brown font indicates no mouse or rat data in swisspalm for the reviewed gene or unreviewed homologs.** **Blue font is used if there is no mouse, rat, or human data in swisspalm for the reviewed gene or unreviewed homologs.** Metabolic isozymes of interest are listed together and studies identifying both or either isozyme are counted.

Gene name	Description	Classification	S-acyl proteomic studies	Targeted studies	Targeted studies citations
ANK2	ankyrin 2	Adaptor	7/48	YES	Gupta *et al*., 2023
BICD1	BICD cargo adaptor 1	Adaptor	0/48	0	
BICD2	BICD cargo adaptor 2	Adaptor	1/48	0	
BICDL1	BICD family like cargo adaptor 1	Adaptor	0/45	0	
BICDL2	BICD family like cargo adaptor 2	Adaptor	0/48	0	
CCDC88A	coiled-coil domain containing 88A	Adaptor	0/45	0	
CCDC88B	coiled-coil domain containing 88B	Adaptor	0/45	0	
CCDC88C	coiled-coil domain containing 88C	Adaptor	0/45	0	
CRACR2A	calcium release activated channel regulator 2A	Adaptor	0/45	0	
HAP1	huntingtin associated protein 1	Adaptor	0/48	0	
HOOK1	hook microtubule tethering protein 1	Adaptor	1/48	0	
HOOK2	hook microtubule tethering protein 2	Adaptor	1/48	0	
HOOK3	hook microtubule tethering protein 3	Adaptor	0/45	0	
HTT	huntingtin	Adaptor	1/48	YES	Huang et al., 2004; Yanai et al., 2006; Huang et al., 2009; Saleem et al.[1], 2010; Singaraja *et al*., 2011; Huang et al., 2011; Sutton et al., 2013; Lin *et al*., 2015; Sanders *et al*., 2015; Lemarié *et al*., 2021; Lemarié *et al*., 2023
KIFAP3	kinesin associated protein 3	Adaptor	1/45	0	
MAPK8IP3	mitogen-activated protein kinase 8 interacting protein 3	Adaptor	2/48	0	
NDE1	nudE neurodevelopment protein 1	Adaptor	0/48	YES	Shmueli *et al*., 2010
NDEL1	nudE neurodevelopment protein 1 like 1	Adaptor	0/48	YES	Shmueli *et al*., 2010
NIN	ninein	Adaptor	0/45	0	
NINL	ninein like	Adaptor	0/45	0	
NUMA1	nuclear mitotic apparatus protein 1	Adaptor	6/48	0	
PAFAH1B1	platelet activating factor acetylhydrolase 1b regulatory subunit 1	Adaptor	6/48	0	
RAB11FIP3	RAB11 family interacting protein 3	Adaptor	1/45	0	
RAB45	RAS and EF-hand domain containing	Adaptor	0/45	0	
RILP	Rab interacting lysosomal protein	Adaptor	0/45	0	
SNAPIN	SNAP associated protein	Adaptor	0/48	0	
SNX5	sorting nexin 5	Adaptor	1/48	0	
SNX6	sorting nexin 6	Adaptor	3/48	0	
SPDL1	spindle apparatus coiled-coil protein 1	Adaptor	1/48	0	
TRAK1	trafficking kinesin protein 1	Adaptor	0/45	0	
TRAK2	trafficking kinesin protein 2	Adaptor	0/25	0	
SPTAN1	spectrin alpha, non-erythrocytic 1	Adaptor	15/48	0	
SPTBN1	spectrin beta, non-erythrocytic 1	Adaptor	14/48	0	
SPTBN2	spectrin beta, non-erythrocytic 2	Adaptor	4/48	0	
ACTB	actin beta	Dynactin	15/48	0	
ACTR1A	actin related protein 1A	Dynactin	7/48	0	
ACTR10	actin related protein 10	Dynactin	4/48	0	
CAPZA1	capping actin protein of muscle Z-line subunit alpha 1	Dynactin	13/48	0	
CAPZA2	capping actin protein of muscle Z-line subunit alpha 2	Dynactin	6/48	0	
CAPZA3	capping actin protein of muscle Z-line subunit alpha 3	Dynactin	0/48	0	
CAPZB	capping actin protein of muscle Z-line subunit beta	Dynactin	16/48	0	
DCTN1	dynactin subunit 1	Dynactin	10/48	0	
DCTN2	dynactin subunit 2	Dynactin	2/48	0	
DCTN3	dynactin subunit 3	Dynactin	1/48	0	
DCTN4	dynactin subunit 4	Dynactin	3/48	0	

DCTN5	dynactin subunit 5	Dynactin	2/48	0	
DCTN6	dynactin subunit 6	Dynactin	1/48	0	
DYNC1H1	dynein cytoplasmic 1 heavy chain 1	Dynein	17/48	0	
DYNC1I1	dynein cytoplasmic 1 intermediate chain 1	Dynein	4/48	YES	Kang *et al* ., 2008
DYNC1I2	dynein cytoplasmic 1 intermediate chain 2	Dynein	4/48	0	
DYNC1LI1	dynein cytoplasmic 1 light intermediate chain 1	Dynein	4/48	0	
DYNC1LI2	dynein cytoplasmic 1 light intermediate chain 2	Dynein	4/48	0	
DYNLL1	dynein light chain LC8-type 1	Dynein	5/48	0	
DYNLL2	dynein light chain LC8-type 2	Dynein	4/48	0	
DYNLRB1	dynein light chain roadblock-type 1	Dynein	0/48	0	
DYNLRB2	dynein light chain roadblock-type 2	Dynein	1/48	0	
DYNLT1	dynein light chain Tctex-type 1	Dynein	1/48	0	
DYNLT3	dynein light chain Tctex-type 3	Dynein	1/48	0	
KLC1	kinesin light chain 1	Kinesin	5/48	0	
KLC2	kinesin light chain 2	Kinesin	2/48	0	
KLC3	kinesin light chain 3	Kinesin	0/48	0	
KLC4	kinesin light chain 4	Kinesin	2/48	0	
CENPE	centromere protein E	Kinesin	1/48	0	
KIF11	kinesin family member 11	Kinesin	4/48	0	
KIF12	kinesin family member 12	Kinesin	0/45	0	
KIF13A	kinesin family member 13A	Kinesin	0/45	0	
KIF13B	kinesin family member 13B	Kinesin	1/48	0	
KIF14	kinesin family member 14	Kinesin	2/48	0	
KIF15	kinesin family member 15	Kinesin	1/48	0	
KIF16B	kinesin family member 16B	Kinesin	0/48	0	
KIF17	kinesin family member 17	Kinesin	1/48	0	
KIF18A	kinesin family member 18A	Kinesin	0/45	0	
KIF18B	kinesin family member 18B	Kinesin	2/48	0	
KIF19	kinesin family member 19	Kinesin	0/45	0	
KIF1A	kinesin family member 1A	Kinesin	4/48	0	
KIF1B	kinesin family member 1B	Kinesin	4/48	0	
KIF1C	kinesin family member 1C	Kinesin	0/48	0	
KIF20A	kinesin family member 20A	Kinesin	3/48	0	
KIF20B	kinesin family member 20B	Kinesin	0/48	0	
KIF21A	kinesin family member 21A	Kinesin	2/48	0	
KIF21B	kinesin family member 21B	Kinesin	0/48	0	
KIF22	kinesin family member 22	Kinesin	1/48	0	
KIF23	kinesin family member 23	Kinesin	4/48	0	
KIF24	kinesin family member 24	Kinesin	0/45	0	
KIF25	kinesin family member 25	Kinesin	0/22	0	
KIF26A	kinesin family member 26A	Kinesin	0/45	0	
KIF26B	kinesin family member 26B	Kinesin	0/45	0	
KIF27	kinesin family member 27	Kinesin	0/48	0	
KIF28P	kinesin family member 28, pseudogene	Kinesin	1/48	0	
KIF2A	kinesin family member 2A	Kinesin	3/48	0	
KIF2B	kinesin family member 2B	Kinesin	0/48	0	
KIF2C	kinesin family member 2C	Kinesin	1/48	0	
KIF3A	kinesin family member 3A	Kinesin	0/48	0	
KIF3B	kinesin family member 3B	Kinesin	1/48	0	
KIF3C	kinesin family member 3C	Kinesin	0/48	0	
KIF4A	kinesin family member 4A	Kinesin	3/48	0	
KIF4B	kinesin family member 4B	Kinesin	0/22	0	
KIF5A	kinesin family member 5A	Kinesin	4/48	0	
KIF5B	kinesin family member 5B	Kinesin	7/48	0	
KIF5C	kinesin family member 5C	Kinesin	3/48	0	
KIF6	kinesin family member 6	Kinesin	0/22	0	

KIF7	kinesin family member 7	Kinesin	0/48	0	
KIF9	kinesin family member 9	Kinesin	0/48	0	
KIFC1	kinesin family member C1	Kinesin	1/48	0	
KIFC2	kinesin family member C2	Kinesin	0/45	0	
KIFC3	kinesin family member C3	Kinesin	0/45	0	
STARD9	StAR related lipid transfer domain containing 9	Kinesin	0/45	0	
HK1	hexokinase 1	Metabolic enzyme	15/48	YES	Chen *et al*., 2022
GPI	glucose 6-phosphate isomerase	Metabolic enzyme	21/48	0	
PFKM/PFKL/PFKP	phosphofructokinase	Metabolic enzyme	33/48	0	
ALDOA/ALDOC	aldolase a & aldolase c	Metabolic enzyme	22/48	0	
TPI1	triosephosphate isomerase	Metabolic enzyme	21/48	0	
GAPDH	glyceraldehyde 3-phosphate dehydrogenase	Metabolic enzyme	21/48	YES	Yang *et al*., 2005
PGK1	phosphoglycerate kinase 1	Metabolic enzyme	21/48	0	
PGAM1	phosphoglycerate mutase 1	Metabolic enzyme	15/48	0	
ENO1/ENO2	enolase alpha & enolase gamma	Metabolic enzyme	20/48	0	
PKM	pyruvate kinase m	Metabolic enzyme	18/48	0	
NMNAT2	nicotinamide mononucleotide adenylyltransferase 2	Metabolic enzyme	0/48	YES	Mayer et al., 2010; Lau et al., 2010; Milde, Gilley, and Coleman, 2013 ; Milde and Coleman, 2014; Niu *et al*., 2020
LDHA/LDHB	lactate dehydrogenase a & lactate dehydrogenase b	Metabolic enzyme	24/48	YES	Chen *et al*., 2024

[1]Saleem AN, Chen Y-H, Baek HJ, Hsiao, Y-W, Huang H-W, Kao H-J, Liu K-M Shen L-F, Song I-W, Tu C-P D, Wu J-Y, Kikuchi T, Justice MJ, Yen JJY, and Chen Y-T (2010) Mice with alopecia, osteoporosis, and systemic amyloidosis due to mutation in Zdhhc13, a gene coding for palmitoyl acyltransferase. PLoS Genetics 6:e1000985.
